# Quantifying the Role of Self-Declared Obstacles to Unachieved Fertility: Proposing A New Method

**DOI:** 10.1007/s10680-025-09747-5

**Published:** 2025-09-24

**Authors:** Qi Cui, Diederik Boertien, Albert Esteve

**Affiliations:** 1 Department of Methodology, London School of Economics and Political Science, London, UK; 2 Centre d’Estudis Demogràfics, Barcelona, Spain; 3 Department of Sociology, Universitat Autònoma de Barcelona, Barcelona, Spain

**Keywords:** Obstacle-removed$\text{TFR}$, Local effect, Spillover effect, Fertility

## Abstract

The discrepancies between desired and actual fertility rates are one of the key topics in fertility studies. This paper aims to explore the fertility gap—the difference between desired fertility size and actual fertility outcomes, and how this gap can be decomposed according to the importance of various perceived barriers to fertility. This article introduces an innovative approach to quantify the impact of removing obstacles that individuals report prevent them from having a child (or another child) on the total fertility rate ($\text{TFR}$). On the one hand, this method offers an alternative perspective on the relationship between the desired number of children and observed fertility outcomes. Unlike conventional analyses that begin with the differences between desired and actual fertility levels, this approach considers that the sum of the fertility gap—defined by the reported reasons that hinder individuals from having children—and the observed fertility level represents the obstacle-removed $\text{TFR}$. On the other hand, this method provides a cause-deleted analysis for fertility, addressing a gap in formal demographic analysis which has historically focussed on mortality research. Although this approach introduces some assumptions, the results offer insights into the relative importance of reported obstacles to fertility.

**Supplementary Information:**

The online version contains supplementary material available at 10.1007/s10680-025-09747-5.

## Background

The exploration of differences between desired and actual fertility rates is a topic that has been examined since foundational analyses by scholars such as Becker (1960), Easterlin (1975), and Bongaarts (1993). These pioneering works were important in elucidating fertility decision-making processes when the fertility rates were relatively high. However, their applicability appears somewhat constrained in contemporary contexts of low fertility, where desired family sizes often exceed the number of children that individuals actually have (Beaujouan & Berghammer, 2019; Bongaarts, 2001). These studies highlight the need to revisit the fertility intention and outcome model to illuminate underlying complexities and social dynamics in fertility behaviours, as the divergence between fertility intentions and actual outcomes becomes increasingly apparent.

It is now widely acknowledged that fertility intentions do not consistently predict actual fertility outcomes (Berrington, 2004; Ní Bhrolcháin & Beaujouan, 2019). Research has also shown that fertility preferences are dynamic rather than fixed over the life course (Liefbroer, 2009). Moreover, when to have children is increasingly associated with changes in the desired age at first childbearing rather than strict adherence to fixed plans (Verweij et al., 2020). The predominant theoretical framework, the Theory of Planned Behaviour (TPB, Ajzen, 1991), is criticised for its inadequate application in fertility analysis, as it fails to address the fertility gap—the difference between desired fertility size (or fertility intentions) and actual outcomes. These intentions are subject to frequent revisions due to changing personal circumstances and external factors, thus complicating the collection of accurate and consistent data (Ajzen & Klobas, 2013).

This paper proposes that the fertility gap—the difference between desired and observed fertility—can be re-conceptualised as a reflection of the barriers that prevent individuals from achieving their fertility potential. Rather than simply comparing survey measures of desired fertility with observed outcomes, our approach takes a counterfactual perspective: it calculates what level of fertility would be realised if the removing of obstacles to fertility that respondents report would result in childbearing. In doing so, we reposition the observed fertility from the left to the right side of the equation, which allows us to view “desired fertility” as an indicator of the obstacle-removed fertility level. This framework shifts the focus from measuring fertility desires directly to understanding and quantifying how self-reported obstacles could influence the potential births. If people report that certain obstacles prevent them from currently having a child, one could consider hypothetical scenarios where these obstacles are removed. This leads to the formulation: *Desired Fertility Size* = *Fertility Gap* + *Fertility Outcome*. This equation suggests that the desired fertility size could serve as an indicator of the level of fertility achievable without barriers. This perspective offers several advantages. First, it provides an alternative method to quantify the ideal fertility level, avoiding the inaccuracies inherent in traditional survey questions about fertility intention or desired fertility size. Second, it provides a quantitative approach towards comparing the relative perceived importance of different barriers towards achieving certain levels of fertility. Third, this approach contributes to formal fertility analysis; similar to cause-deleted life table analysis in mortality studies, a comparable methodology in fertility analysis is presently lacking.

Despite these advantages, quantifying an obstacle-free fertility analysis poses three main challenges. First, data have to be obtained on the obstacles that people experience towards having children. In our case, data are available on which obstacles people themselves perceive as preventing them from having children. Second, the reasons inhibiting fertility behaviours are often interconnected and may not directly translate into immediate fertility behaviours. For example, once financial barriers are eliminated, other issues such as health or relationship difficulties might emerge, delaying or preventing people from having children. Quantifying the importance of different obstacles therefore is more indicative of the relative importance of various obstacles and provides an upper-bound estimated of “obstacle-free” fertility for counterfactual situations where only one obstacle is removed. Third, fertility is a recurrent event that varies by birth order, hence removing a barrier that prevents the next birth (i.e. the $\left(i+1\right)$ th birth) may increase the likelihood of additional births beyond the next one (i.e. $\left(i+2\right)$ th, $\left(i+3\right)$ th, $\left(i+4\right)$ th births, etc.). We define the effect of removing a barrier on the next birth as the “local effect”, while we refer to the effect on all higher-order births as the “spillover effect”. These challenges can be partially addressed through improved survey measurements and by making assumptions about the interdependencies of fertility barriers and their immediate effects on fertility behaviour, which will be detailed later in this manuscript. Therefore, this paper aims to answer a critical research question: To what extent does total fertility increase after hypothetically removing obstacles to fertility at various birth orders (local effect) and across higher birth orders (spillover effect)?

To illustrate this method, data from the 2018 Spanish Fertility Survey will be used, which asked, “Why have you not had any children?” with 20 fixed options and one open-ended option. Such data are not always available, but more datasets are emerging such as the second wave of the Social Networks and Fertility module (2021) from the Longitudinal Internet for Social Science panel, which includes a question on: “Can you tell us more about what makes you (un)certain about whether or not to have children?” (open-ended question) (Xu et al., 2022).

In the following sections, the methodological framework will be introduced, presenting two key assumptions and their rationales alongside three equations. Each equation will be illustrated with a simulated example to clarify and visualise their implications. The method will then be applied to the data from the 2018 Spanish Fertility Survey, demonstrating several what-if scenarios. Finally, the approach and its limitations will be discussed.

## Method

The goal of our method is to calculate a counterfactual level of fertility if obstacles that people report prevent them from having children were to be removed. Two essential assumptions are interconnected in this analysis. First, the reported obstacles are assumed to be independent of each other and unrelated by birth order (Assumption 1). This means that obstacles stemming from material reasons, for instance, are considered unrelated to the presence or absence of other types of obstacles like those stemming from partner-related issues. Second, it is assumed that the reported obstacles are the sole factors preventing individuals from having a child (or more children) (Assumption 2). In other words, if individuals identify lacking financial support as the primary barrier, then mitigating or eliminating financial barrier is hypothesised to correspondingly increase the likelihood of childbirth.

**Fig. 1:**
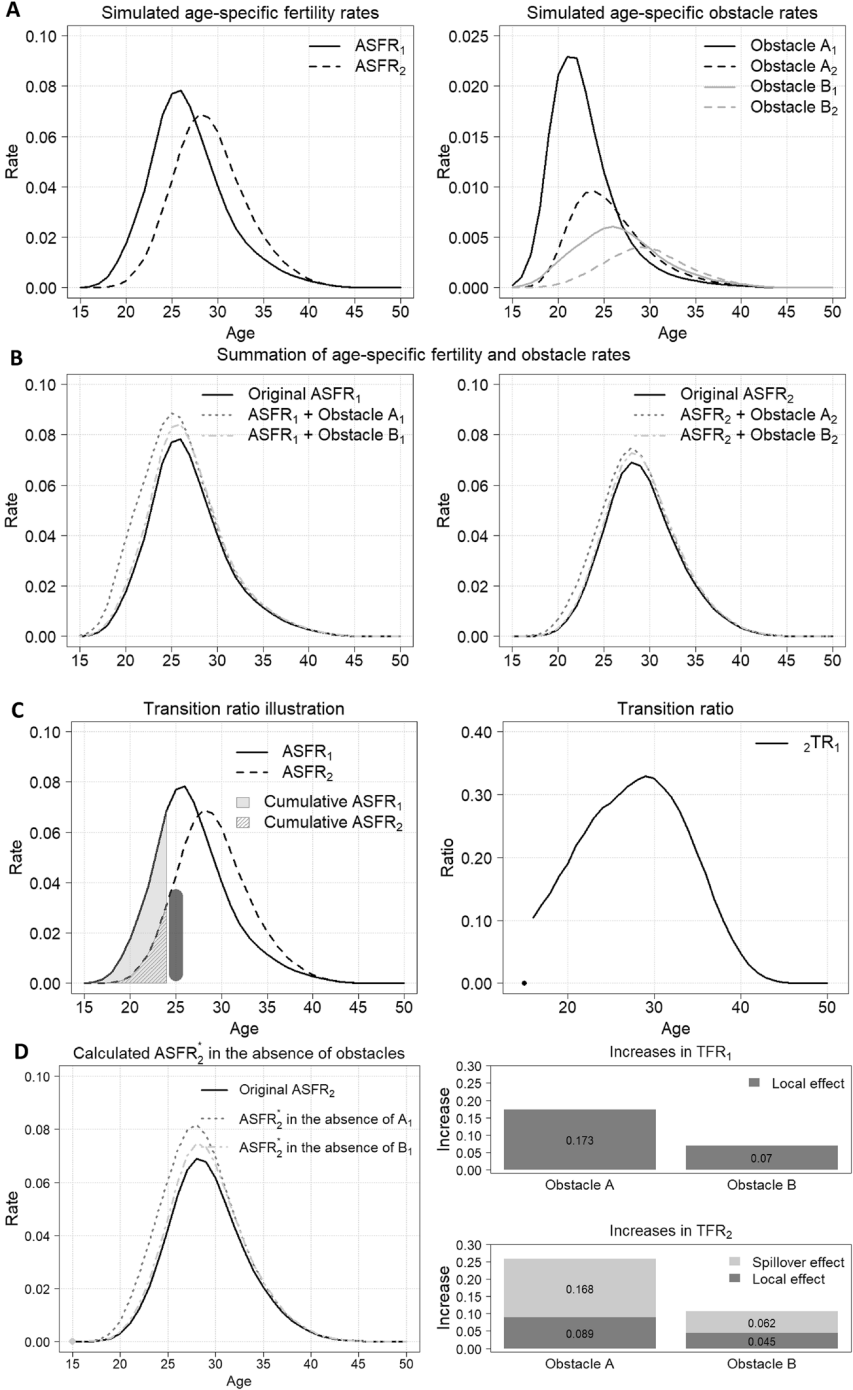
Simulated cohort fertility and obstacles data. *Notes*: Calculated by authors based on Eqs. (1)–(3)

A simulated cohort dataset is used to demonstrate this approach. It includes the first- and the second- ordered age-specific fertility rates (labelled as ${\text{ASFR}}_{1}$ and ${\text{ASFR}}_{2}$) and two different obstacles that impede women having the first and the second birth (obstacles ${A}_{1}$ and ${B}_{1}$ for the first birth and obstacles ${A}_{2}$ and ${B}_{2}$ for the second birth). Panel A of Fig. 1 shows two age-specific fertility rates and the obstacle rates. The ${\text{TFR}}_{1}$ and ${\text{TFR}}_{2}$ are 0.732 and 0.659, respectively. And the modal ages of the first- and second-order age-specific fertility rates are 26.16 and 28.78 year old, correspondingly.

In Fig. 1, two simulated sets of cohort data are presented: the age-specific fertility rate and the age-specific obstacle rate by birth order. The age-specific obstacle rate is defined as the number of women, at each age, who report a particular obstacle that stops them from having a birth divided by the number of women at risk of giving a birth. Thus, in this analysis, obstacles are assumed to have a binary effect (if reported, they fully prevent a birth). In our example, obstacle A has a larger effect than obstacle B because a higher proportion of women report it: its total obstacle rates are 0.173 versus 0.070 for the first birth and 0.089 versus 0.045 for the second birth, respectively. These figures indicate that a larger proportion of women report obstacle A, and it tends to D impact women at their younger ages (modal ages: 21.93 vs. 26.41 for the first birth and 24.21 vs. 29.62 for the second birth). Based on the two assumptions, if the obstacle that stops women having $i$ th birth at age $x$, ${O}_{i}\left(x\right)$ is removed, then the obstacle-free $i$ th-order age-specific fertility rate, ${\text{ASFR}}_{i}^{*}\left(x\right)$, will be,

1 $${\text{ASFR}}_{i}^{*}\left(x\right)={\text{ASFR}}_{i}\left(x\right)+{O}_{i}\left(x\right) .$$

The *local effect* on ${\text{TFR}}_{i}$ will be ${\text{TFR}}_{i}^{*}-{\text{TFR}}_{i}={\sum }_{x=\alpha }^{x=\beta }{O}_{i}\left(x\right),$ where $\alpha , \beta$ are the minimum and maximum female reproductive ages, accordingly. Therefore, the summation of the birth order $\text{ASFR}$ s and obstacle rates can be calculated based on Eq. (1), which are shown in Panel B of Fig. 1. Equation (1) can be partially relaxed with respect to the first assumption that all obstacles are independent by multiplying age‐specific weights $\delta \left(x\right)$ to indicate only part of the obstacles may contribute to a birth. In the current formulation, Eq. (1) uses $\delta (x)=1$, assuming that all obstacles contribute to the birth with maximum potential (more details see Discussion and Supplementary Information 2). In other words, we produce upper-bound estimates of how high fertility would be if obstacles were to be removed.

Fertility data are ordered by birth, implying that eliminating the barriers preventing the first birth might naturally facilitate subsequent second and third births (i.e. spillover effect). To quantify the effect of removing reason on the higher birth order, the following two formulae are developed based on the nature of cohort age-specific fertility rate and on the obstacles distribution.

Here, the concept of a transition ratio is introduced, which is defined as,

2 $${{}_{i+1}\text{TR}}_{i}\left(x\right)=\frac{{\text{ASFR}}_{i+1}\left(x\right)}{\sum_{\alpha }^{x-1}{\text{ASFR}}_{i}\left(a\right)-\sum_{\alpha }^{x-1}{\text{ASFR}}_{i+1}\left(a\right)}, \text{for} \,\, x>\alpha$$

$${{}_{i+1}\text{TR}}_{i}\left(x\right)=0, \text{for} \,\, x=\alpha$$

where $x$ and $a$ symbolise age. $a=\alpha , \alpha +1, \alpha +2, \dots , x-1$. $i$ is the birth order. The denominator is the proportion of women who have experienced the $i$ th birth but not yet the $\left(i+1\right)$ th birth by age $x-1$, and accordingly, Eq. (2) indicates the probability that a woman aged $x$, having had the $i$ th birth, will have the $\left(i+1\right)$ th birth within an average span of 1 year. Therefore, $1-{{}_{i+1}\text{TR}}_{i}\left(x\right)$ gives the complementary probability, indicating the probability that women who have given the $i$ th birth at age $x-1$ but do not give the $\left(i+1\right)$ th birth at age $x$.

To visualise the transition ratio, an example is illustrated in Fig. 1, Panel C (left plot). The ${{}_{2}\text{TR}}_{1}\left(25\right)$ is calculated as follows, $\frac{\text{dark grey bar}}{\text{grey area} - s\text{triped area}}$, where grey area is the proportion of women who have experienced their first birth by age 24. The striped area represents the proportion of women who have experienced their second birth by age 24. Thus, the difference between these two areas defines the proportion of women who have experienced their first birth but not the second by age 24. The dark grey bar represents the proportion of women who give the second birth at age 25. Therefore, the ratio of the dark grey bar to $\left(\text{grey area} - \text{striped area}\right)$ determines the probability that a woman aged 25, having had the first birth, will have the second birth within an average span of 1 year.

The right plot of Panel C in Fig. 1 shows the calculated transition ratio based on the simulated ${\text{ASFR}}_{1}$ and ${\text{ASFR}}_{2}$. The transition ratio from the first birth to the second starts from 0.104, increases gradually, and reaches the peak at age 29.65. After that, the probability having the second birth decreases among those who have given the first birth in the previous ages.

Note that the range of ${{}_{i+1}\text{TR}}_{i}\left(x\right)$ should fall between 0 and 1. However, when applied to period data, which are synthetic measures that combine information across different cohorts, the calculated transition ratios may sometimes fall outside this range. In such cases, values are adjusted to 0 if negative or 1 if exceeding 1. Equation (2) can be extended to analyse the transition ratio for higher birth orders, like ${{}_{3}\text{TR}}_{1}\left(x\right)$ and ${{}_{4}\text{TR}}_{1}\left(x\right)$. A more general formulation is introduced in SI 2. Similarly, Eq. (2) can be generalised by incorporating an age‐specific proportion, $\theta \left(x\right)$, to capture more realistic scenarios of variation in the strength of fertility desires across age groups/the life course. In the present analysis, Eq. (2) assumes $\theta \left(x\right)=1$, meaning that all women desire further births (more details see Discussion and Supplementary Information 2).

With Eqs. (1) and (2), the new *obstacle-free* age-specific fertility rate can be defined as,

3 $${\text{ASFR}}_{i+1}^{*}\left(x\right)={{}_{i+1}\text{TR}}_{i}\left(x\right)\sum_{k=\alpha }^{x-1}\left\{{\text{ASFR}}_{i}^{*}\left(k\right)\prod_{j=k+1}^{x-1}\left[1-{{}_{i+1}\text{TR}}_{i}\left(j\right)\right]\right\}, \quad \text{for} x<\beta$$

$${\text{ASFR}}_{i+1}^{*}\left(\beta \right)={{}_{i+1}\text{TR}}_{i}\left(\beta \right)\sum_{k=\alpha }^{\beta -1}{\text{ASFR}}_{i}^{*}\left(k\right)\prod_{j=k+1}^{\beta -1}\left[1-{{}_{i+1}\text{TR}}_{i}\left(j\right)\right]+\left[{\text{ASFR}}_{i}^{*}\left(\beta -1\right)+{\text{ASFR}}_{i}^{*}\left(\beta \right)\right]{{}_{i+1}\text{TR}}_{i}\left(\beta \right), \quad \text{ for} x=\beta$$

where $x$, $k$, and $j$ all represent age, operating at various levels of the equation. $k=\alpha , \alpha +1, \alpha +2, \alpha +3, \dots , x-1$ and $j=k+1,k+2,k+3, \dots , x-1$. Note that when $k=x-1$, the product, $\prod_{j=x}^{x-1}\left[1-{{}_{i+1}\text{TR}}_{i}\left(j\right)\right]$, with index $j$ goes from $x$ to $x-1$, is an empty product. By convention, the value of an empty product is considered 1. Thus, ${\text{ASFR}}_{i+1}^{*}\left(x\right)={\text{ASFR}}_{i}^{*}\left(x-1\right){{}_{i+1}\text{TR}}_{i}\left(x\right)$. When $x=\alpha$, the starting age is the minimum reproductive age, we set the new ${\text{ASFR}}_{i+1}^{*}\left(\alpha \right)=0$. Making this definition is reasonable, as the pregnancy lasts for about 40 weeks. And, from the current birth to the following birth other factors can also extend the period, like the duration of postpartum infecundability, coitus frequency, and menstrual cycle. The spillover effect on ${\text{TFR}}_{i+1}$ will be ${\text{TFR}}_{i+1}^{*}-{\text{TFR}}_{i+1}={\sum }_{x=\alpha }^{x=\beta }\left[{\text{ASFR}}_{i+1}^{*}\left(x\right)-{\text{ASFR}}_{i+1}\left(x\right)\right]$.

For illustration of Eq. (3), consider an example with $i=1$, $\alpha =15$, $\beta =50$, and $x=17$. ${\text{ASFR}}_{2}^{*}\left(17\right)={{}_{2}\text{TR}}_{1}\left(17\right){\text{ASFR}}_{1}^{*}\left(15\right)\left[1-{{}_{2}\text{TR}}_{1}\left(16\right)\right]+{{}_{2}\text{TR}}_{1}\left(17\right){\text{ASFR}}_{1}^{*}\left(16\right)$. The first part gives the increment contributed by women who have given first birth at age 15, ${\text{ASFR}}_{1}^{*}\left(15\right)$, and did not give the second birth at age 16, by multiplying $1-{{}_{2}\text{TR}}_{1}\left(16\right)$, but might give the second birth at age 17, by multiplying ${{}_{2}\text{TR}}_{1}\left(17\right)$. While the second part gives the increment made by women who have given the first birth at age 16, ${\text{ASFR}}_{1}^{*}\left(16\right)$, and might give the second birth at age 17, by multiplying ${{}_{2}\text{TR}}_{1}\left(17\right)$. These two parts are quite intuitive, as the woman who can give the second birth at age $x$, are only those who have given the first birth before age $x$. In an extreme example where all ${{}_{i+1}\text{TR}}_{i}s$ are equal to 1 (implying every woman who has the $i$ th child will also have the $\left(i+1\right)$ th birth in the following year). Then, Eq. (3) can be re-organised as ${\text{ASFR}}_{i+1}^{*}\left(x\right)={{}_{i+1}\text{TR}}_{i}\left(x\right){\text{ASFR}}_{i}^{*}\left(x-1\right)={\text{ASFR}}_{i}^{*}\left(x-1\right), for x<\beta$, and ${\text{ASFR}}_{i+1}^{*}\left(\beta \right)={\text{ASFR}}_{i}^{*}\left(\beta -1\right)+{\text{ASFR}}_{i}^{*}\left(\beta \right), for x=\beta$. Thus, the operation of Eq. (3) indeed forces the curve to shift to the right by one age step (see Fig. S1 in SI 1).

Two advantages of Eq. (3) should be highlighted. One advantage is that since the obstacles are independent (Assumption 1), thus the spillover effect on ${\text{TFR}}_{i+1}$ from combined multiple obstacles will be the sum of spillover effects from individual obstacles. The underlying mathematical proof is straightforward, we do not present it here. Another advantage is that the total spillover effects at $\left(i+1\right)$ th will be always lower than the local effects at $i$ th birth, as the transition ratio is almost always lower than 1, which ensures ${\text{ASFR}}_{i}^{*}\left(x\right)$ can partially transfer to the higher birth orders. This property aligns with observed reality, as not all women who have given the $i$ th birth would give the $\left(i+1\right)$ th birth.

Using the same example, ${\text{ASFR}}_{2}^{*}$ s in the absence of obstacles ${A}_{1}$ and ${B}_{1}$ are calculated based on Eq. (3) and presented in the left plot of Panel D in Fig. 1. Compared with the original ${\text{ASFR}}_{2}$, removing obstacles ${A}_{1}$ and ${B}_{1}$ both lead to the increases in ${\text{TFR}}_{2}$, especially obstacle ${A}_{1}$. However, removing obstacles ${A}_{1}$ forces the ${\text{ASFR}}_{2}^{*}$ to younger ages (from 28.78 to 28.35), while removing obstacles ${B}_{1}$ forces the ${\text{ASFR}}_{2}^{*}$ to older ages (from 28.78 to 28.79).

## Data

As discussed in the introduction, the 2018 Spanish Fertility Survey is one of the surveys that recorded the reasons why respondents said they did not have a (or another) child. The 2018 Spanish Fertility Survey is cross-sectional and required respondents to rank the three main obstacles from a provided list. To apply the approach using the Spanish dataset, several assumptions must be introduced. First, it is assumed that the first reported reason is the most significant and the only obstacle preventing women from achieving their desired fertility level. Consequently, if this obstacle were removed, it is assumed that women would immediately transition to a higher parity. Second, the cross-sectional data are treated as cohort data. Because the survey is cross-sectional, all age-specific fertility and obstacle rates are period measures; in our analysis we treat them as if they trace the life course of one synthetic cohort. Thus, the proportion reported at age $x$ is interpreted as the share of that hypothetical cohort who have experienced the event (first birth, second birth, or specific obstacle) by age $x$. By doing so, more fluctuations and negative values over ages are inevitably introduced into the analysis. In the results section, these fluctuations will not be smoothed out but instead, negative values will be set to zero, as negative age-specific birth and reason rates do not exist and including them could lead to uninterpretable results.

The 2018 Spanish Fertility Survey is a representative survey conducted by the Spanish National Statistics Institute (INE, 2019). The survey interviewed 14,566 women and 2619 men born between 1962 and 2000, ages 18–55. Besides demographic information (e.g. age, sex, education, occupation, partnership, religion, etc*.*), detailed fertility histories have been recorded, and respondents are asked to indicate why they did not (yet) have (more) children (chosen from a list of 21 options). In this study, women’s birth histories were re-constructed based on the year of the survey and the information on their biological children’s birthdates. Since the data do not allow us to determine whether migrants gave birth in Spain, and since they would merit separate analysis for which there are too few cases (1748 non-native Spanish women), migrants are excluded from this study. The final sample consists of 12,808 native-born Spanish women. The 21 obstacles that women reported as reasons for not (yet) achieving their desired number of children were re-grouped into six categories, including partner, health and material obstacles, do not want to have (more) children, not ready to have (more) children, and others (for details, see SI 3). Discussing a specific obstacle out of 21 is beyond the main focus of our research.

Our goal is to quantify how important different obstacles towards achieving desired fertility goals are. In this counterfactual analysis, we therefore do not consider not wanting or not feeling ready to have a child as an obstacle towards fertility in the “obstacle-free” counterfactual scenarios. This decision follows Régnier-Loilier et al. (2011) and treats deliberate non-childbearing as a personal preference, not an external barrier. These people are therefore considered to not give birth at a given age even in the counterfactual scenario where all obstacles were to be removed. The original survey questions used to classify obstacles are provided in Supplementary Information (SI 3). It should be noted that the survey asks the participants to provide a maximum of three obstacles in order of preference. To avoid the issue of overcounting, focus on the first of the three obstacles was mentioned. 54.84, 18.92, and 26.24% of women provided one reason, two reasons, and three reasons, respectively. To provide less fluctuated and more reliable results, only the first three births are taken into account, because less than 7 per cent of women have given a third birth and most of them do not want to give the fourth birth.

## Results

The approach was applied to the 2018 Spanish Fertility Survey. Initially, the distribution of all obstacles alongside the age-specific fertility rates, obstacle rates, and transition ratios was displayed. A table summarising the estimates with 95% confidence intervals is then presented, along with another table that includes $\text{TFR}$ estimates, obstacle-removed $\text{TFR}$, and desired fertility sizes (an indicator estimated from the survey).

**Fig. 2:**
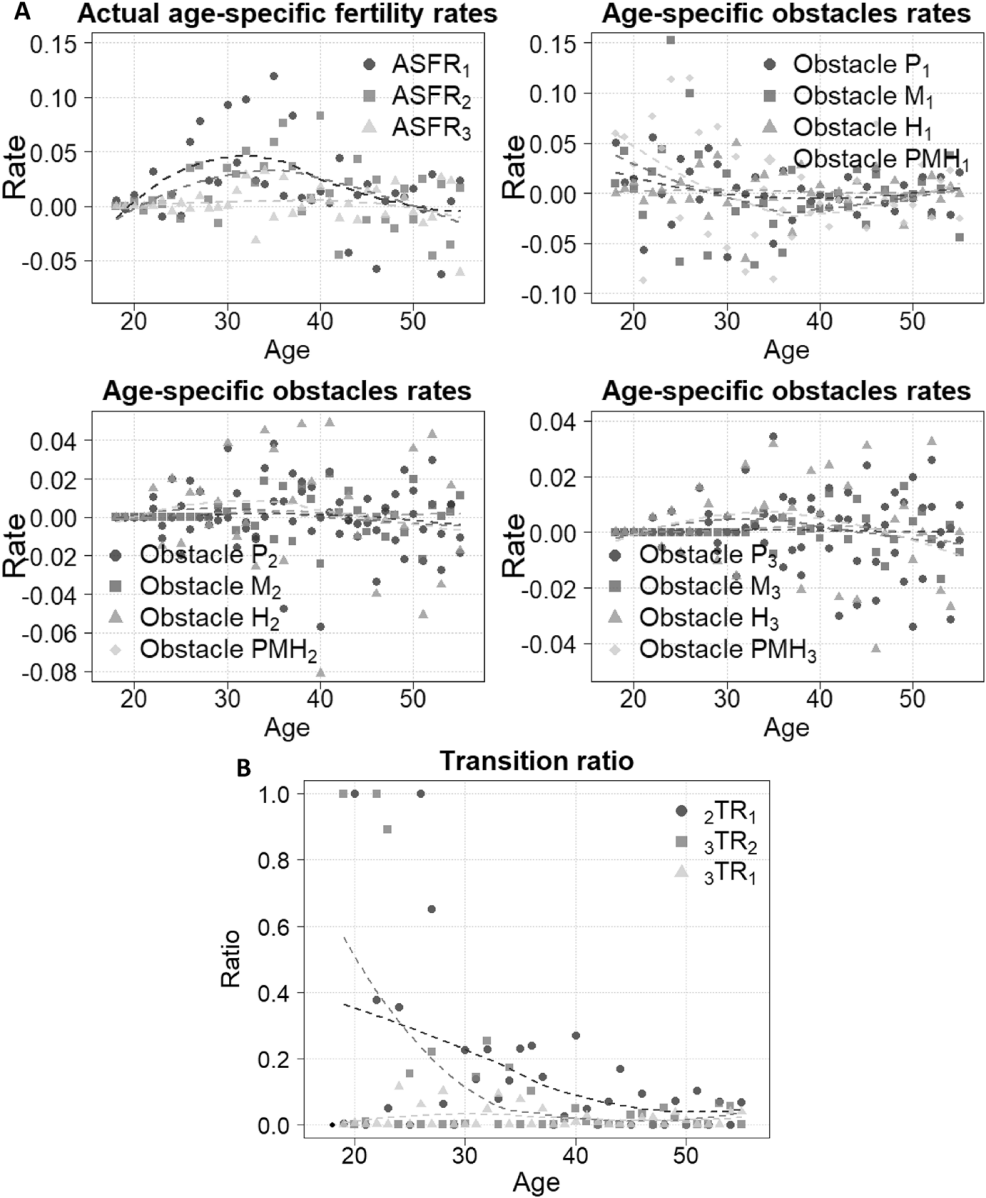
Estimated fertility and obstacles rates (Panel **A**) and transition ratio (Panel **B**). *Source*: Calculated by authors based on the 2018 Spanish Fertility Survey. *Notes*: P, M, H, and PHM represent partner, material, health, and the combination of these three reasons, separately. The sub-index indicates birth order or the reasons that stop women achieving the specific birth order. For visualisation purposes, the dash lines in Panels A and B are LOESS-smoothed curves (span = 1.0)

Panel A of Fig. 2 illustrates the age-specific fertility rate by birth order. Despite fluctuations, first births are more common than second births, and the number of third births is almost negligible. Additionally, the first birth occurs at younger ages compared with subsequent births. In Panel A, age-specific obstacle rates are included. It is evident that the major reported reasons impeding women from achieving their desired family size are material (M), followed by partner (P) and health-related (H) reasons. A combination of these three obstacles (PMH) is also provided. It should be noted that all the age-specific fertility and obstacle rates exhibit significant fluctuation. Due to the synthetic cohort from a cross-sectional survey, there are instances where the calculated obstacle rates become negative (i.e. the cumulative proportion at a given age is lower than that of an earlier age). Since negative obstacle rates are not meaningful in practice, we force these negative values to zero to ensure the calculations remain interpretable. Panel B presents the transition ratio based on Eq. (2). The probability of transitioning to a higher parity from a lower one at younger ages is considerably higher than at older ages. The transition ratios ${}_{3}{\text{TR}}_{1}$ and ${}_{3}{\text{TR}}_{2}$ are significantly lower than ${}_{2}{\text{TR}}_{1}$ across most ages, except for early ages, indicating that having a second child is much more common than having a third among the women who have given the first birth.

Table 1 summarises the local and spillover effects on $\text{TFR}$ by birth order. For ${\text{TFR}}_{2}$ and ${\text{TFR}}_{3}$, the spillover effect is the dominant effect. Overall, the material-related obstacles play the dominant role. After removing material-related obstacles from the analysis of the three births, the total fertility rate would increase by 1.12 births, followed by partner reasons (0.82 births) and health-related reasons (0.74 births). Table 2 presents six summarising indicators: the first four are the estimated total fertility rate by birth order, the all-obstacle-removed $\text{TFR}$, denoted as ${\text{TFR}}^{*}$, and the desired fertility sizes. It should note that the last two indicators (i.e. ${\text{TFR}}^{*}$ and the survey-based desired fertility sizes) demonstrate quite similar levels. This similarity does not apply to the general conclusion that the obstacle-free total fertility is comparable to desired fertility size, because the calculations for obstacle-removed total fertility (as demonstrated above) are more complicated than a simple survey question.

**Table 1:** Estimated local and spillover effects with 95% confidence intervals by different obstacles

		Partner	Material	Health	All combined*
Increases in ${\text{TFR}}_{1}$	Local effect	0.0539	0.0234	0.0601	0.1374
		[0.0532, 0.0546]	[0.0229, 0.0239]	[0.0593, 0.0609]	[0.1362, 0.1385]
Increases in ${\text{TFR}}_{2}$	Local effect	0.0047	0.0257	0.0522	0.0827
		[0.0046, 0.0049]	[0.0252, 0.0263]	[0.0514, 0.0529]	[0.0818, 0.0835]
	Spillover effect (from the first birth)	0.3355	0.5013	0.2537	0.5654
		[0.3312, 0.3398]	[0.4953, 0.5072]	[0.2494, 0.2581]	[0.5603, 0.5704]
Increases in ${\text{TFR}}_{3}$	Local effect	0.0124	0.0357	0.0209	0.0690
		[0.0120, 0.0128]	[0.0351, 0.0363]	[0.0205, 0.0213]	[0.0682, 0.0698]
	Spillover effect (from the first birth)	0.2059	0.2837	0.1453	0.3741
		[0.2023, 0.2095]	[0.2790, 0.2884]	[0.1422, 0.1483]	[0.3686, 0.3795]
	Spillover effect (from the second birth)	0.2049	0.2490	0.2056	0.2770
		[0.2017, 0.2081]	[0.2455, 0.2524]	[0.2023, 0.2088]	[0.2734, 0.2805]
Summary	Increases in ${\text{TFR}}_{1}$	0.0539	0.0234	0.0601	0.1374
		[0.0532, 0.0546]	[0.0229, 0.0239]	[0.0593, 0.0609]	[0.1362, 0.1385]
	Increases in ${\text{TFR}}_{2}$	0.3402	0.5270	0.3059	0.6480
		[0.3359, 0.3446]	[0.5210, 0.5330]	[0.3014, 0.3104]	[0.6429, 0.6532]
	Increases in ${\text{TFR}}_{3}$	0.4232	0.5683	0.3717	0.7200
		[0.4170, 0.4295]	[0.5611, 0.5756]	[0.3658, 0.3776]	[0.7122, 0.7278]
	Increases in $\text{TFR}$	0.8174	1.1188	0.7377	1.5054
		[0.8091, 0.8257]	[1.1080, 1.1295]	[0.7296, 0.7459]	[1.4950, 1.5159]

*Source*: Calculated by authors based on the 2018 Spanish Fertility Survey

*Notes*: The 95% confidence interval is based on 1000 bootstrap samples. As discussed in the Method section, the overall combined spillover effect should equal the sum of all individual obstacles. However, the statistics calculated here differ from this sum because we applied different normalisation processes. For each individual obstacle, we directly set negative values to zero. In contrast, for the combined obstacles, we first sum each individual obstacle, which may turn some negative values positive, and then set negative values to zero. The spillover effect on the increase in ${\text{TFR}}_{3}$ from the first birth is calculated using Equation S1 to construct ${{}_{3}\text{TR}}_{1}$ and Eq. (3). In doing so, we set the first two age-specific fertility rates to zero (i.e. ${\text{ASFR}}_{3}^{*}\left(18\right)={\text{ASFR}}_{3}^{*}\left(19\right)=0$) to represent 2 years of zero fertility

**Table 2:** Estimated total fertility rates by birth orders, obstacle-free total fertility rates, and desired family sizes with 95 confidence intervals

	${\text{TFR}}_{1}$	${\text{TFR}}_{2}$	${\text{TFR}}_{3}$
Estimated	0.7485	0.4938	0.0674
95% CI	[0.7470, 0.7499]	[0.4921, 0.4955]	[0.0666, 0.0682]

	$\text{TFR}$	${\text{TFR}}^{*}$	Desired family sizes
Estimated	1.3097	2.8151	2.6969
95% CI	[1.3066, 1.3128]	[2.8047, 2.8255]	[2.6583, 2.7354]

*Source*: Calculated by authors based on the 2018 Spanish Fertility Survey

*Notes*: ${\text{TFR}}^{*}$ is calculated based on the absence of the combination of material, partner and heath related issues for all birth orders. The 95% confidence interval is based on 1000 bootstrap samples

## Discussion

This paper proposes a new method for formal fertility analysis based on two assumptions. One assumption is that the reported obstacles are independent of each other. Another assumption is that these reported obstacles are the sole factors preventing individuals from having a child (or more children). This approach offers a new perspective for quantifying the perceived relative importance of reported obstacles on the $\text{TFR}$. The results present various what-if scenarios, which can serve as a reference for to understand the relative weight the population gives to groups of obstacles towards achieving desired fertility. From a methodological perspective, four points need to be highlighted.

First, removing an obstacle can increase fertility not only at the next birth order, referred to as the “local effect” (i.e. the $\left(i+1\right)$ th birth), but also at subsequent higher birth orders, known as the “spillover effect” (i.e. the $\left(i+2\right)$ th, $\left(i+3\right)$ th, etc.). As shown in Eq. (3), this spillover effect is more nuanced because not all gains from the local effect are transferred to higher orders. A simpler yet naïve approach might assume that all higher-parity lines shift in direct proportion to the local effect, which would double-count births and result in a bimodal fertility schedule (for instance, implying that some women have multiple second births in 1 year). Panel D of Fig. 1 and Table 1 illustrates how the method presented here avoids this unrealistic outcome by separately accounting for local and spillover effects.

Second, this method has broader implications. As shown in Table 2, the method offers an alternative way to interpret the gap between fertility outcomes and perceived desired fertility sizes. Although it derives from self-reported survey data, this approach differs from traditional methods that rely solely on direct questions about fertility intentions or desires. By focussing on obstacles that respondents themselves identify, it aims to quantify a hypothetical “obstacle-free” $\text{TFR}$, thereby shedding light on how specific obstacles might shape fertility outcomes. By adjusting the equations listed above, the analysis can be adapted to describe more realistic scenarios. For instance, if there is strong empirical evidence, a theoretical argument, or a practical reason suggesting that only part of the age-specific obstacle rate can convert to births (i.e. not all obstacles removed at each age would lead to births), the calculation in Eq. (1) can be directly modified by applying age-specific weights $\delta \left(x\right)$ or a fixed weight $\delta$ for all ages. The rest of the calculations remain unchanged. In this context, both hypothetical and Spanish results assume $\delta =1$. This assumption implies that removing obstacles would maximumly increase fertility rates without any other correlated impediments preventing individuals from having children. Additionally, the transition ratio calculation can be extended to yield more realistic results by multiplying by the age-specific proportion, $\theta \left(x\right)$, which represents the proportion of women desiring to have a higher-order birth, as not all women wish to have a second (or subsequent) birth(s). In Eq. (2), $\theta \left(x\right)$ is set to 1, meaning that all women are assumed to have the potential to progress to the next births, regardless of whether they have achieved their desired fertility level. Future work could refine this by estimating $\theta \left(x\right)$ from survey responses based on whether a woman has achieved her desired fertility level, yielding a more realistic “obstacle-free” fertility scenario. The equations incorporating $\delta \left(x\right)$ and $\theta \left(x\right)$ are detailed in SI 2.

Third, the method provides greater generalisability and can be easily extending to analyse other types of sequential events. For instance, to assess the impact of changes in marriage rates on first-order marital fertility, the transition ratio can be adapted to describe the transition from marriage to first-order marital birth. Subsequently, Eq. (3) reallocates the hypothetical marriage proportions across ages to compute the marital fertility rate. While a recent study by Nishikido et al. (2022) offers a simplistic method for quantifying the effects of marriage age composition on first-order marital fertility, it assumes that all married women will invariably give first birth. This assumption contrasts with the method here, which calculates potential births based on probability. Additionally, their method has to depend on extrapolation for the initial and final marriage proportions, potentially restricting the extrapolation scope. Such an approach could suggest hypothetical first-order fertility rates exceeding 1, which is impossible since these rates should inherently be less than 1.

Fourth, compared to other formal demographic analyses (Feeney & Yu, 1987; Bongaarts, 1990, 2001; Chiang, 1968; Preston et al. 2001), this approach is unique and innovative. The transition ratio is similar to the classic parity progression ratio (PPR; see SI 4 for a detailed discussion). Following the standard PPR (PPR) concept (Preston et al., 2001, p. 104), the age-specific parity progression ratio (ASPPR) is defined as the ratio of the number of women who have had their $\left(i+1\right)$ th birth at a specific age $x$ to the number of women who have had their $i$ th birth at that same age. It measures the proportion of women at a given age and parity who progress to the next parity within the *same* year. In contrast, the transition ratio measures the probability that a woman aged $x$, having had the $i$ th birth at age $x$ but not the $\left(i+1\right)$ th birth previously, will have the $\left(i+1\right)$ th birth in the *next* year and age. The transition ratio combines with the lower birth order obstacle-free $\text{ASFRs}$ to calculate the higher birth order obstacle-free $\text{ASFR}$s, as described in Eq. (3). In contrast, the $\text{ASPPR}$ can be directly applied to construct the $\text{TFR}$ (Preston et al., 2001, p.104).

The model proposed by Bongaarts (2001) established the connection between actual $\text{TFR}$ and desired fertility size via six proximate variables, including unwanted births, sex preferences, child mortality, the tempo effect, involuntary family limitation, and competing preferences. This method, instead, focuses on the obstacles collected in the survey questions, such as material obstacles and partner issues, and removes non-negative intention obstacles (Régnier-Loilier et al., 2011). It thereby estimates $\text{TFR}$s as the relative contribution of these obstacles to the gap between observed and desired fertility. The mathematical logic differs from the Bongaarts model, which uses a multiplicative function to adjust $\text{TFR}$s uniformly across all $\text{ASFR}$ s, whereas our approach builds on $\text{ASFR}$ s. One of the assumptions align with those in the cause-deleted life table analysis (Andersen et al., 2013; Chiang, 1968; Preston et al., 2001), which presumes that removing obstacles or causes will affect changes in $\text{TFR}$ or life expectancy at subsequent ages, correspondingly. Regarding the differences between our fertility analysis and the cause-deleted mortality analysis, while the latter removes specific causes of death to calculate potential increases in life expectancy, the fertility method focuses on removing obstacles that inhibit achieving desired fertility levels. However, births, unlike deaths, are not inevitable events, even when considered by birth order. This shift from mortality to fertility introduces distinct analytical challenges as presented in method section and necessitates additional assumptions, due to the need to account for varying birth orders.

Despite offering a novel way to quantify the impact of removing perceived obstacles on fertility, our approach has several important limitations. First, removing an obstacle does not guarantee that an additional birth will occur. Fertility decisions are influenced by many factors (e.g. relationship quality, timing, and unforeseen life risks) and each of these can change over time. In Eqs. (1) and (2), where $\delta \left(x\right)=1$ and $\theta \left(x\right)=1$, we are essentially modelling a maximum potential effect under assumptions that all obstacles are independent, and that every woman who reports a stopping-birth obstacle would be most likely to have further children if it were removed. Thus, our estimates should be viewed as hypothetical “what-if” scenarios, rather than direct predictions of actual birth outcomes. Even if a specific barrier—such as unstable employment—were removed, many young adults might still postpone parenthood until additional conditions are met, or simply because they do not yet wish to become parents (Datta et al., 2023). Consequently, policies that focus solely on improving youth employment are unlikely to convert one-to-one into births; any fertility response is more likely to be modest or delayed.

Second, the assumption that all obstacles are independent can lead to overestimates of how much total fertility might rise if multiple obstacles were removed. In reality, obstacles often interact or reinforce each other. For instance, health complications can trigger financial burdens, or partner disagreements might intersect with job instability. Our extended versions of Eqs. (2) and (3), which include $\delta \left(x\right)$ and $\theta \left(x\right)$, have been introduced to account for partial correlation and the possibility that not everyone progresses to another birth. However, these additions alone cannot fully capture the complexity of interdependent reasons. In reality, decisions around childbearing depend on changing life circumstances and deeper personal preferences, some of which may not be captured in a single survey question. Third, cross-sectional 2018 Spanish Fertility Survey data were used to reconstruct age-specific fertility and obstacle patterns by treating them as a single cohort’s experience. Such an approach can introduce fluctuations and biases in the resulting estimates, which are partly managed by setting negative values to zero. However, final results should be viewed as indicative. Fourth, subjective reporting of obstacles may be affected by recall errors or respondents’ emotional states, as the survey’s fixed list of obstacles and its request for up to three main reasons might not capture the full complexity of individual decision-making.

## Conclusion

The paper introduces an innovative method to quantify the impact of removing the reasons that are perceived to prevent women from having a child (or another child) on the total fertility rate ($\text{TFR}$). This method offers an alternative perspective on the relationship between the desired number of children and observed fertility outcomes. Conventionally, the analysis of the fertility gap starts from the differences between desired and actual levels. The new approach posits that the sum of the fertility gap (measured by the reasons that impede people from giving birth) and the observed fertility level equates to the obstacle-removed $\text{TFR}$. Additionally, it provides a cause-deleted analysis for fertility, which fills one of the gaps in formal demographic analysis, where cause-deleted analysis was predominantly focussed on mortality research. Although this approach has generated some potential issues, the results demonstrate the importance of applying this method, providing what-if scenarios. These scenarios not only contribute to academic discussions but also offer valuable insights for policymakers aiming to understand and potentially mitigate the barriers that the population perceive towards achieving desired fertility levels.

## Supplementary Information

Below is the link to the electronic supplementary material.

Supplementary file1 (DOCX 269 kb)
