# Supplementary material for: Quantifying the Role of Self-Declared Obstacles to Unachieved Fertility: Proposing A New Method
Source: Eur J Popul. 2025 Sep 24;41(1):24. doi: 10.1007/s10680-025-09747-5 (PMC12460207; doi:10.1007/s10680-025-09747-5)
Supplement: Supplementary file 1 — Supplementary file1 (DOCX 269 kb) [file 10680_2025_9747_MOESM1_ESM.docx]

## **Supplementary information**

### **SI 1. Visualisation**


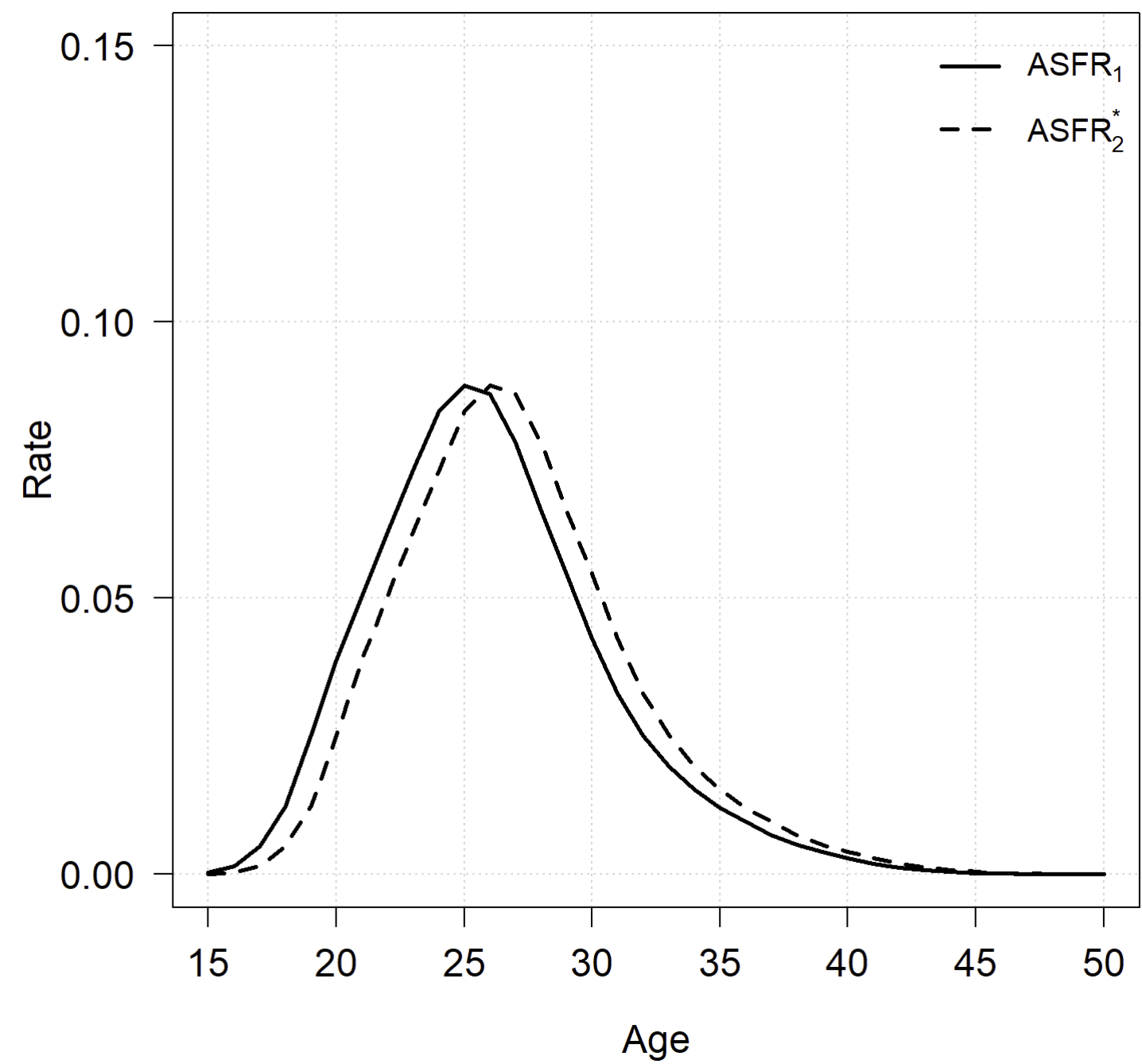


**Fig. S1** Obstacle-free age-specific fertility rate based on ${{}_{2}{TR}}_{1}\left( x \right)=1$. *Source*: Simulated data.

The same simulated dataset as presented in the Methods section is used. ${ASFR}_{2}^{*}\left( x \right)$ is calculated based on Eq. (3), where${{}_{2}{TR}}_{1}\left( x \right)=1$. As shown in Fig. S2, ${ASFR}_{2}^{*}\left( x \right)$ shifts to the right by one age step compared to ${ASFR}_{1}\left( x \right)$. This shift indicates that women who give birth for the first time will have their second child one year later.

### **SI 2. Method extension**

For the transition over two birth orders, we have,

$${{}_{i+\tau}{TR}}_{i}\left( x \right)={{}_{i+\tau}{TR}}_{i}\left( x \right)$$

$$=\frac{{ASFR}_{i+\tau}\left( x \right)}{\sum_{\alpha}^{x-i-\tau+1} {ASFR}_{i}\left( a \right)-\sum_{\alpha}^{x-i} {ASFR}_{i+\tau}\left( a \right)}, for x\geq\alpha+i+\tau-1 \left( S1 \right)$$

$${{}_{i+\tau}{TR}}_{i}\left( x \right)=0. for \alpha\leq x<\alpha+i+\tau-1$$

Equation (S1) ensures at least $\tau$ years birth interval from the $i$th birth to the ($i+\tau$)th birth, resulting in a zero transition ratio from the initial birth at age $\alpha$ up to aged $\alpha+i+\tau-2$.

It should note that Equation S1 does not does not assume that women must have the $\left( i+1 \right)$th birth before achieving higher order births (e.g., $\left( i+2 \right)$th birth). Instead, it treats $\left( i+1 \right)$th and higher order births without imposing a strict sequential order (as indicated by the denominator in Equation S1 and the accompanying example). This approach helps separate impacts of removing obstacles on the $\left( i+1 \right)$th birth and on higher order births independently.

For instance, let $i=1$, $\alpha=15$, $\tau=2$, and $x=17$, the transition ratios, ${{}_{3}{TR}}_{1}\left( 15 \right)$, ${{}_{3}{TR}}_{1}\left( 16 \right)$, and ${{}_{3}{TR}}_{1}\left( 17 \right)$ are calculated as,

$${{}_{3}{TR}}_{1}\left( 15 \right)={{}_{3}{TR}}_{1}\left( 16 \right)=0$$

$${{}_{3}{TR}}_{1}\left( 17 \right)=\frac{{ASFR}_{3}\left( 17 \right)}{{ASFR}_{1}\left( 15 \right)-\left[ {ASFR}_{3}\left( 15 \right)+{ASFR}_{3}\left( 16 \right) \right]}$$

The denominator of ${{}_{3}{TR}}_{1}\left( 17 \right)$ is the proportion of women who have experienced their first birth by age 15 but not yet their third birth by age 16. Thus, ${{}_{3}{TR}}_{1}\left( 17 \right)$ indicates the probability that a woman aged 17, having had her first birth at age 15, will have her third birth by age 17. Since the first birth occurs at age 15, a two-year interval is necessary before the third birth can occur, leading to zero values for${{}_{3}{TR}}_{1}\left( 15 \right)$ and ${{}_{3}{TR}}_{1}\left( 16 \right)$.

As discussed above, the realistic version of Equations (1) and (2) are shown below,

$${ASFR}_{i}^{**}\left( x \right)={ASFR}_{i}^{*}\left( x \right)\delta\left( x \right)$$

$$=\left[ {ASFR}_{i}\left( x \right)+O_{i}\left( x \right) \right]\delta\left( x \right), \left( S2 \right)$$

and,

$${}_{i+1}{{TR}_{i}^{**}}\left( x \right)={{}_{i+1}{TR}}_{i}\left( x \right)\theta\left( x \right)$$

$$=\frac{\theta\left( x \right){ASFR}_{i+1}\left( x \right)}{\sum_{\alpha}^{x-1} {ASFR}_{i}\left( a \right)-\sum_{\alpha}^{x-1} {ASFR}_{i+1}\left( a \right)}, for x>\alpha\left( S3 \right)$$

$${}_{i+1}{{TR}_{i}^{**}}\left( x \right)=0. for x=\alpha$$

If $\delta\left( x \right)=\theta\left( x \right)=1$, Equations (S2) and (S3) reduce to the original Equations (1) and (2), respectively.

### **SI 3. Re-classification**

The original survey questions:

7.5.1 You would have liked or would like to have children?

1 Yes

6 No 🡪 go to the question 7.5.3

7.5.2. How many?

|__|__|

7.5.3. Why have you not had any children? (select max. 3 options in order of preference)

1 I could not get pregnant or carry a pregnancy to term

2 I have not had a partner or they was not suitable

3 I don't want to be a mother

4 I wanted to continue studying

5 Health problems or discomfort.

6 Pregnancies, deliveries and caring for the children are hard for women

7 Too young to have children

8 Too old to have children

9 Would conflict with my professional career

10 Lack of economic resources

11 Bad housing conditions

12 Excess of work in the home

13 Lack or shortage of infant schools

14 Because of work (own or partner)

15 Fear that the child would be born with health problems

16 Losing freedom and not having time for other activities

17 Because of the concerns and challenges of raising children

18 Difficulty in reconciling work and family life

19 My partner didn't want any

20 I don't like the current model of society for a child.

21 Others Please explain in detail_____________

We re-grouped the 21 reasons in to 6 categories.

Do not want to:

3 I don't want to be a mother

6 Pregnancies, deliveries and caring for the children are hard for women

16 Losing freedom and not having time for other activities

Not ready

4 I wanted to continue studying

7 Too young to have children

Partner

2 I have not had a partner or they were not suitable

19 My partner didn't want any

Health

1 I could not get pregnant or carry a pregnancy to term

5 Health problems or discomfort.

8 Too old to have children

15 Fear that the child would be born with health problems

Material

9 Would conflict with my professional career

10 Lack of economic resources

11 Bad housing conditions

12 Excess of work in the home

13 Lack or shortage of infant schools

14 Because of work (own or partner)

18 Difficulty in reconciling work and family life

Others

17 Because of the concerns and challenges of raising children

20 I don't like the current model of society for a child.

21 Others reasons


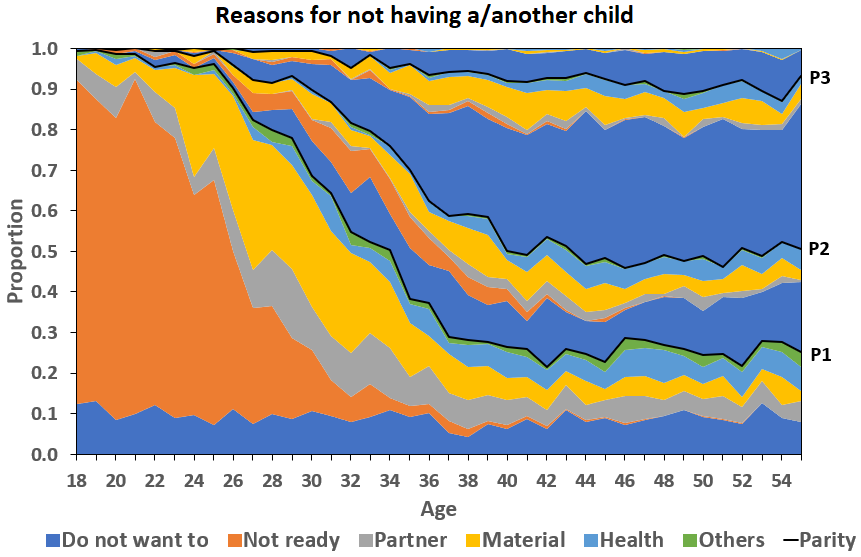


**Fig. S2** Reasons for not having a or another child.

*Source*: Calculated by authors based on the 2018 Spanish Fertility Survey.

*Note*: In a synthetic cohort, P1, P2, and P3 represent the proportions of women who have not yet had their first, second, and third births, respectively. Therefore, (1-P1), (1-P2), and (1-P3) represent the proportions of women who have experienced their first, second, and third births, respectively.

### **SI 4. Comparison with age-specific parity progression ratio**

According to the standard parity progression ratio (PPR) concept (Preston et al., 2001, p.104), the age-specific PPR is defined as the ratio of the number of women who have had their $\left( i+1 \right)$th birth at a specific age $x$ to the number of women who have had their $i$th birth at that same age,

$${{}_{i+1}{ASPPR}}_{i}\left( x \right)=\frac{W_{i+1}\left( x \right)}{W_{i}\left( x \right)} , (S4)$$

where $W_{i+1}\left( x \right)$ is the number $\left( i+1 \right)$th birth to women aged $x$ and $W_{i}\left( x \right)$ is the number of women aged $x$ who have $i$th birth. Both nominator and denominator divide the number women aged $x$, which gives,

$${{}_{i+1}{ASPPR}}_{i}\left( x \right)=\frac{\sum_{\alpha}^{x} {ASFR}_{i+1}\left( a \right)}{\sum_{\alpha}^{x} {ASFR}_{i}\left( a \right)} . (S5)$$

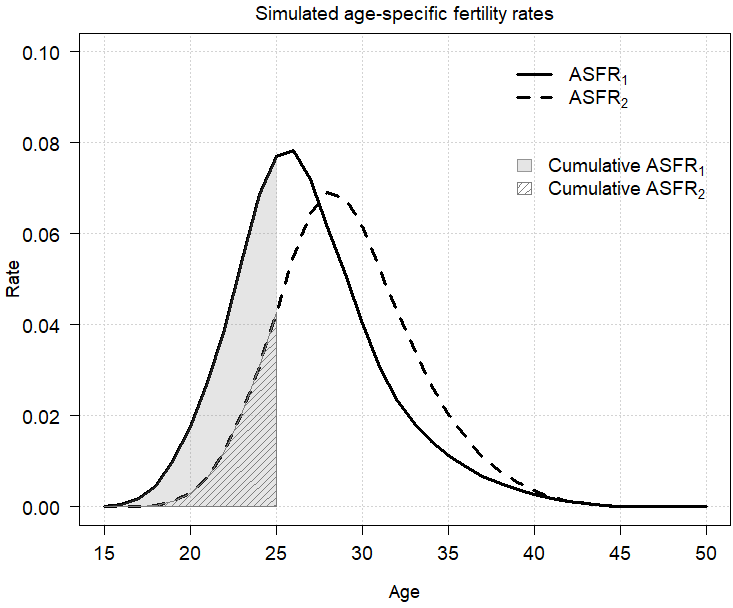


**Fig. S3** Age-specific parity progression ratio visualisation. *Source*: Simulated data.

Figure S3 shows the visualisation of ${{}_{2}{ASPPR}}_{1}\left( 25 \right)$. The denominator (the blue area) of Equation S5 is the cumulative ${ASFR}_{1}\left( 25 \right)$. The nominator of Equation S5 is represented by the green bar, showing the cumulative ${ASFR}_{2}\left( 25 \right)$.
